# Dramatic Response of Nail Psoriasis to Infliximab

**DOI:** 10.1155/2011/107928

**Published:** 2011-05-10

**Authors:** Gilles Safa, Laure Darrieux

**Affiliations:** Department of Dermatology, Centre Hospitalier de Saint-Brieuc, 10, rue Marcel Proust, 22000 Saint-Brieuc, France

## Abstract

Nail psoriasis, affecting up to 50% of psoriatic patients, is an important cause of serious psychological and physical distress. Traditional treatments for nail psoriasis, which include topical or intralesional corticosteroids, topical vitamin D analogues, photochemotherapy, oral retinoids, methotrexate, and cyclosporin, can be time-consuming, painful, or limited by significant toxicities. Biological agents may have the potential to revolutionize the management of patients with disabling nail psoriasis. We present another case of disabling nail psoriasis that responded dramatically to infliximab.

## 1. Introduction

Nail psoriasis, affecting up to 50% of psoriatic patients, is an important cause of distress, impairment of manual dexterity, and pain [1]. Nail involvement varies from pitting to nail dystrophy. Psoriatic nail disease is a therapeutic challenge, and to date, patients and physicians are often dissatisfied with current standard therapeutic approaches. Infliximab, which is approved for the treatment of moderate to severe plaque psoriasis and psoriatic arthritis, is a chimeric monoclonal antibody that inhibits the action of tumour necrosis factor alpha (TNF*α*). Several reports have confirmed its usefulness in treating nail psoriasis [2]. The present paper describes another case of disabling nail psoriasis that responded dramatically to infliximab.

## 2. Case Presentation

**Figure 1:**
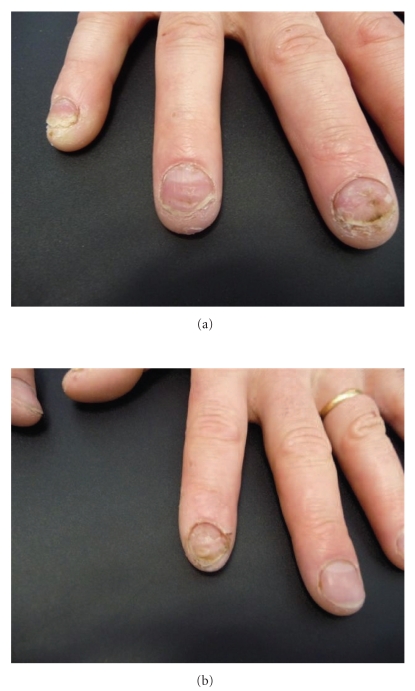
Subungual hyperkeratosis, pitting, and onycholysis of right-hand fingernails 3, 4, and 5 (a) and of left-hand fingernail 2 (b).

A 34-year-old farmer with a history of stable localized plaque psoriasis was referred to our department with psoriatic nail disease involving four fingernails. The toenails did not show any significant lesions, and no symptoms of psoriatic arthritis were noted. He had previously been prescribed topical treatments, including potent topical corticosteroids and vitamin D_3_ analogs, without any efficacy. The patient's discomfort was affecting his ability to work and his quality of life because of psychosocial impairments. On examination, subungual hyperkeratosis, pitting, and onycholysis were seen in four fingernails (Figures 1(a) and 1(b)). Because of the presence of disabling symptoms in this patient, acitretin (25 mg daily) was initially started, but this drug proved ineffective after 6 months of therapy. 

**Figure 2:**
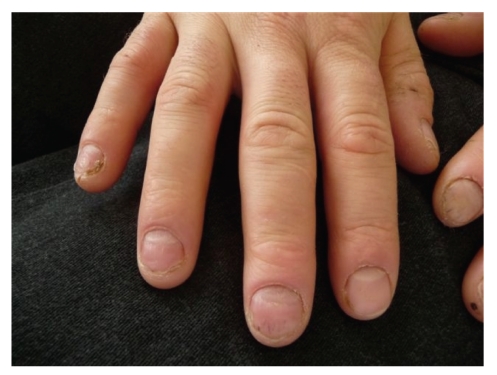
Fingernails after the second infusion of infliximab.

After screening for infection, neoplasm, and autoimmunity, all of which were negative, the patient was started on infliximab at a dose of 5 mg/kg administered at weeks 0, 2, and 6. A dramatic improvement was noticed after the second infusion of infliximab at week 6 (Figure 2). The patient's nails remained free of psoriatic lesions after the third infusion. This treatment regimen was followed by a single infusion every 8 weeks for maintenance therapy.

## 3. Discussion

Biological agents have demonstrated efficacy for the treatment of plaque psoriasis and psoriatic arthritis and are now widely used. Even if the severity of psoriatic skin lesions remains the primary reason to start biological treatments, nail psoriasis should be considered a valid reason to start these therapies because of its large impact on daily living activities and quality of life. Furthermore, nail psoriasis can be a predictor of future inflammatory joint damage, a precursor of psoriatic arthritis, and a visible indicator of disease activity [3]. There are currently no standardized therapeutic regimens for nail psoriasis. Traditional treatments for nail psoriasis, which include topical or intralesional corticosteroids, topical vitamin D_3_ analogues, photochemotherapy, oral retinoids, methotrexate, and cyclosporin, can be time-consuming, painful, or limited by significant toxicities [4]. 

Biological agents that target cytokines may have the potential to revolutionize the management of patients with disabling nail psoriasis. These agents include anti-TNF*α* agents such as infliximab, adalimumab, and etanercept, and anti-interleukin (IL)-12/-23, the first drug of a new class of biotherapy agents.

Adalimumab is a fully human anti-TNF*α* antibody that is administered subcutaneously every 2 weeks. In a prospective, open-label, uncontrolled study conducted in nine European countries, patients with active psoriatic arthritis received adalimumab 40 mg every other week for 12 weeks in addition to their pre-existing antirheumatic treatment. Of 442 patients, 259 had nail involvement. After the relatively short treatment duration of 12 weeks, the median reduction in Nail Psoriasis Severity Index (NAPSI) score was 57%. Clearance of psoriasis of the nails was increasing in those patients who continued adalimumab up to week 20. The NAPSI improvements were independent of other changes in skin assessment measures and occurred regardless of joint response status [5]. 

Etanercept is a TNF*α* receptor fusion protein administered subcutaneously, which binds with and antagonizes the action of TNF*α*. In one post hoc analysis, etanercept 25 mg twice weekly produced a mean reduction in NAPSI score of 51% after 54 weeks in 711 patients with psoriasis, 80% of whom had nail involvement [6]. In another retrospective study of 66 patients treated with etanercept at 25 mg or 50 mg twice weekly at intermittent intervals over a period of 3.4 years, nail involvement improved significantly in each of the treatment cycles [7].

Ustekinumab is a fully human anti-IL-12/-23 monoclonal antibody that binds with high specificity and affinity to the shared p40 protein subunit of the cytokines IL-12 and IL-23, blocking the differentiation and expansion of T-helper (Th)1 and Th17 populations. It has recently been approved in the USA, Europe and Canada for the treatment of moderate to severe plaque psoriasis. Recently, ustekinumab has also been reported as an effective therapeutic alternative in nail psoriasis [8]. 

Infliximab is a chimeric monoclonal antibody that inhibits TNF*α* and is administered intravenously. At the present time, the best evidence for the efficacy of a TNF*α* inhibitor in nail psoriasis comes from a phase III, multicentre, double-blind, placebo-controlled trial designed to evaluate long-term efficacy and safety of infliximab in patients with moderate to severe plaque psoriasis. The primary endpoint of the study was the proportion of patients achieving at least 75% improvement in Psoriasis Area and Severity Index (PASI) compared with baseline. The percentage improvement in NAPSI at weeks 10, 24, and 50 was also specifically investigated. In this well-controlled trial, infliximab resulted in complete clearing of nail psoriasis in 6.9% of patients within 10 weeks, rising to 26.4% after 24 weeks, and 44.7% after 50 weeks. Nail clearance was observed in 1.7% and 5.1% of patients at weeks 10 and 24 in placebo recipients, but this increased to 34.5% at week 38 and to 48.2% at week 50 after the patients had switched to infliximab [2, 9]. Infliximab is one of the most extensively studied biological agents in dermatological practice and is considered by many to be the most effective treatment for nail psoriasis to date [10, 11]. No drug is completely safe, and several safety issues should be considered. However, cumulative evidence indicates that treatment with infliximab is safe and well tolerated, especially if physicians are thoughtful in diagnosing infections and infusion reactions early [12]. In our patient, infliximab showed remarkable and rapid effectiveness in the treatment of psoriatic nail disease. In addition, the present case illustrates the need to further evaluate biological therapies and their cost effectiveness, especially as first-line systemic agents, for the treatment of severe psoriatic nail disease. We believe that infliximab may represent a treatment of choice for the many patients with this distressing condition.
